# Spatial Lipidomics of the Human Prefrontal Cortex for Metabolic Insights in Schizophrenia

**DOI:** 10.1192/j.eurpsy.2026.10248

**Published:** 2026-07-31

**Authors:** P. Enrico, C. Cabasino, G. Delvecchio, P. Brambilla, Y. Torrente, I. Bongarzone

**Affiliations:** 1 Department of Neurosciences and Mental Health; 2 Neurology Unit, Fondazione IRCCS Ca’ Granda Ospedale Maggiore Policlinico; 3 Department of Diagnostic Innovation, Fondazione IRCCS Istituto Nazionale dei Tumori, Milan, Italy

## Abstract

**Introduction:**

Schizophrenia (SCZ) is a severe psychiatric disorder with only partly understood biological mechanisms. Lipids, which account for more than half of the brain’s dry weight, play fundamental roles in myelination, neurotransmission, and immune-glial interactions. Increasing evidence has linked lipid dysregulation to psychiatric disorders, yet most studies rely on bulk lipidomics, which lacks anatomical resolution. Spatial approaches are needed to characterize lipid distribution in the human brain and to investigate disease-related alteration

**Objectives:**

The aim of this study was to perform high-resolution spatial lipidomic mapping of the dorsolateral prefrontal cortex (DLPFC) in post-mortem human brains, and to conduct preliminary analyses of lipid alterations in SCZ compared with matched controls.

**Methods:**

Post-mortem DLPFC tissue was obtained from the NIMH Human Brain Collection Core. Five non-diagnostic cases were analyzed to define the spatial distribution of individual lipid species, while an additional cohort of five SCZ and five matched controls was included for preliminary group comparisons. Sections were processed with a standardized matrix-assisted laser desorption ionization (MALDI) imaging workflow in negative-ion mode (Figure 1). Multivariate segmentation and dimensionality reduction were applied to identify regional molecular profiles. Region- and group-level differences were tested with ANOVA including false discovery rate correction. Lipid assignments were confirmed on tissue by MALDI-LIFT MS/MS and validated through curated database and literature matching of fragment ions.

**Results:**

Spatial analyses revealed reproducible separation of white and grey matter subregions, with PCA showing that the first five components explained 87% of variance (Figure 2). Sulfatides were enriched in white matter, whereas N-acyl phosphatidylserines localized to upper cortical layers, with highly significant regional differences (pFDR < 10⁻¹⁰ for the most discriminant species). Preliminary SCZ versus control analyses based on Pearson’s correlation values and ROC curves stratified by white and grey matter identified phospholipid alterations suggesting possible links with compartment-specific neuroglial and metabolic processes (Figure 3).

**Image 1:**
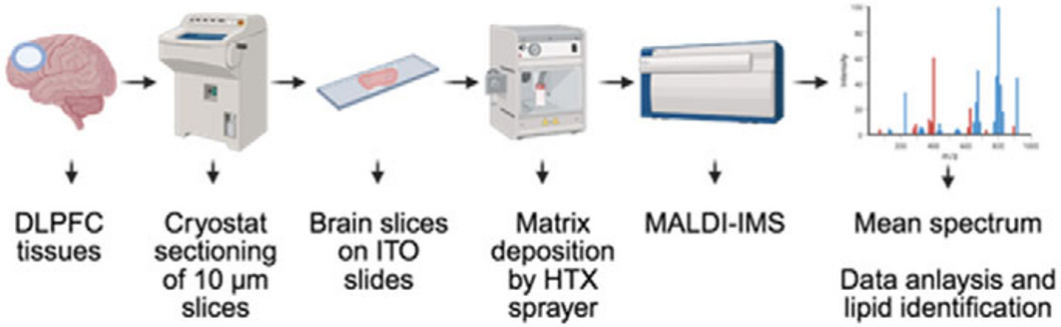
Image 1: Long description.

**Image 2:**
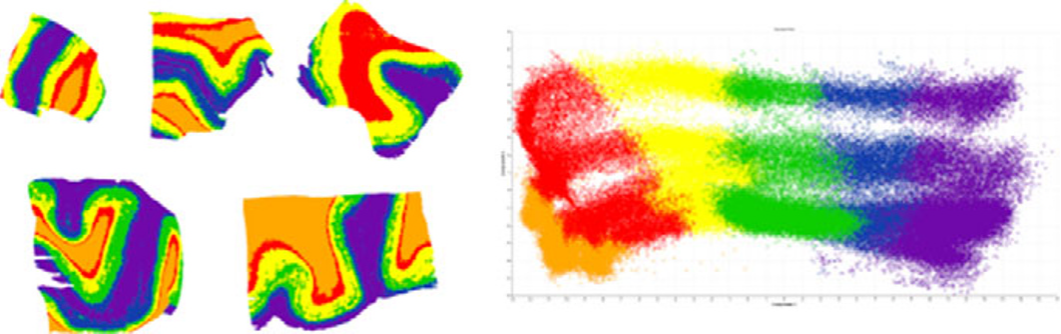
Image 2: Long description.

**Image 3:**
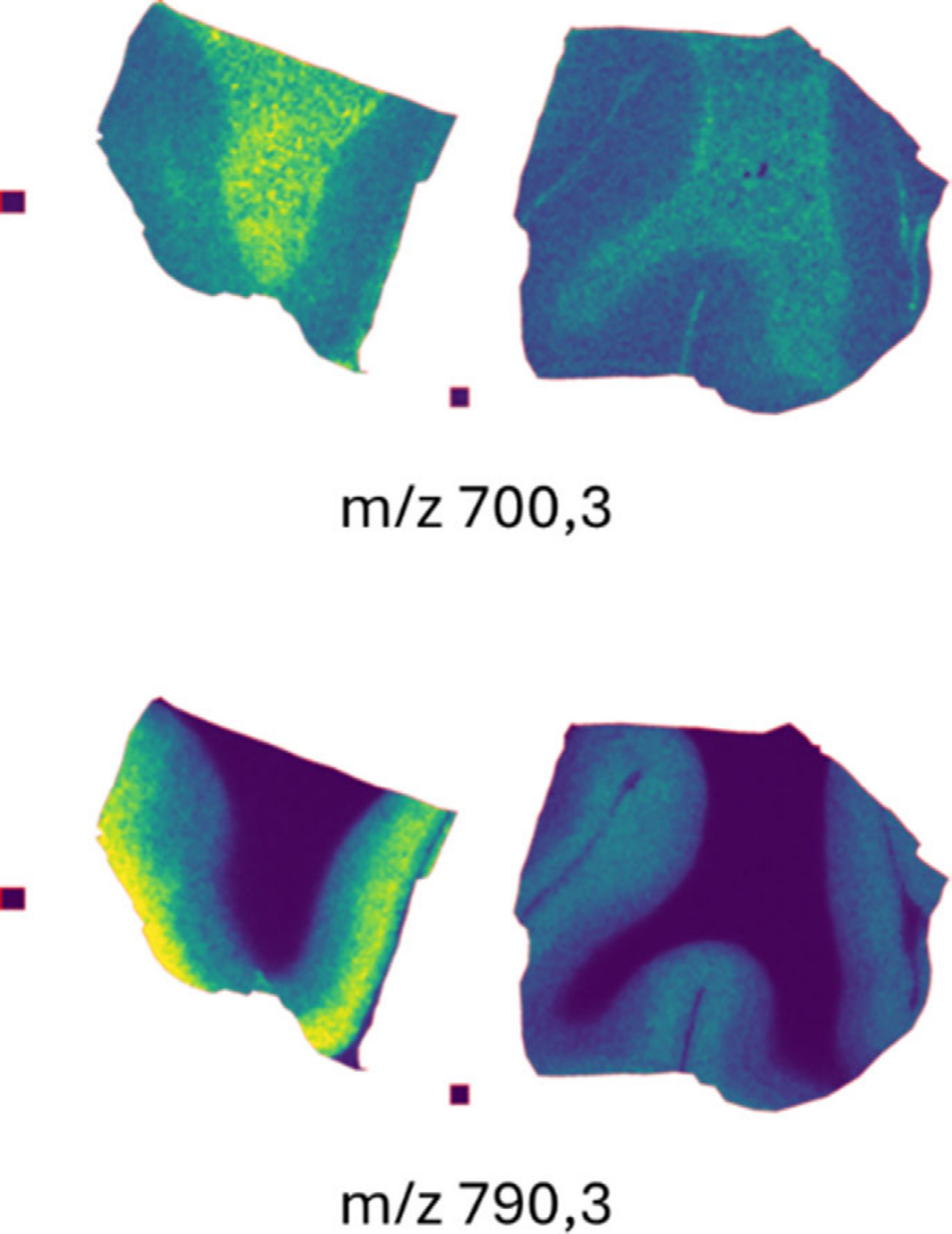
Image 3: Long description.

**Conclusions:**

This work provides one of the first detailed spatial lipidomic mappings of the human DLPFC, uncovering novel region- and layer-specific signatures with potential biological relevance. Preliminary case-control analyses highlight lipid deregulations that may contribute to cortical dysfunction in SCZ. These findings emphasize the translational value of spatial omics approaches for identifying metabolic alterations and candidate biomarkers that could inform precision psychiatry.

**Disclosure of Interest:**

None Declared

## Long descriptions

### Image 1: Long description

The workflow consists of six sequential stages connected by right-pointing arrows.

1. D L P F C tissues. An illustration of a human brain with a highlighted circular region in the frontal lobe.

2. Cryostat sectioning of 10 micrometers slices. An illustration of a laboratory cryostat machine.

3. Brain slices on I T O slides. A glass slide holding a pink tissue section.

4. Matrix deposition by H T X sprayer. An illustration of an automated spraying device.

5. M A L D I I M S. An illustration of a mass spectrometry instrument.

6. Mean spectrum and Data analysis and lipid identification. A bar graph showing Intensity on the y-axis from 0 to 100 and m forward slash z on the x-axis from 0 to 1000. The graph contains red and blue peaks, with a prominent blue peak near 800 m forward slash z.

Navigate back to Image 1:.

### Image 2: Long description

The left panel contains five irregular, fragment-like shapes arranged in two rows. Each shape features a contoured heat map with concentric bands of color. The color gradient follows a consistent sequence: orange at the core, followed by yellow, green, blue, and purple at the outermost edges. The shapes appear to be cross-sections of biological tissue with wavy, folded internal structures.

The right panel is a large scatter plot titled Score Plot. The Y-axis is labeled Component 2 and the X-axis is labeled Component 1. The data points are densely packed and color-coded to match the heat maps in the first panel. From left to right along the X-axis, the data forms distinct but overlapping vertical bands in the following order:

* An orange cluster at the bottom-left.

* A large red cluster spanning the left side.

* A broad yellow cluster in the center-left.

* A green cluster in the center-right.

* A blue cluster in the right-middle.

* A purple cluster on the far right.

The data points are distributed in three main horizontal tiers across the plot, with the highest density in the center and right sections.

Navigate back to Image 2:.

### Image 3: Long description

The figure contains four panels showing spatial distribution of ions in tissue sections.

Top Row: Labeled m forward slash z 700,3.

* The left panel shows a high intensity yellow-green region concentrated in the center of the tissue section, fading to dark blue at the edges.

* The right panel shows a more uniform, lower intensity teal distribution across the entire section with subtle structural outlines.

Bottom Row: Labeled m forward slash z 790,3.

* The left panel shows high intensity yellow-green regions localized specifically at the outer left and right margins of the tissue, with a dark purple low-intensity void in the center.

* The right panel shows a similar pattern where the outer folded structures of the tissue are teal, while the central core is dark purple, indicating an absence of the ion in that region.

Navigate back to Image 3:.

